# Naringin Improves Diet-Induced Cardiovascular Dysfunction and Obesity in High Carbohydrate, High Fat Diet-Fed Rats

**DOI:** 10.3390/nu5030637

**Published:** 2013-02-27

**Authors:** Md. Ashraful Alam, Kathleen Kauter, Lindsay Brown

**Affiliations:** 1 School of Biomedical Sciences, The University of Queensland, Brisbane 4072, Australia; E-Mail: sonaliagun@yahoo.com; 2 Department of Biological and Physical Sciences, The University of Southern Queensland, Toowoomba 4350, Australia; E-Mail: Kate.Kauter@usq.edu.au

**Keywords:** naringin, obesity, hypertension, inflammation, mitochondria

## Abstract

Obesity, insulin resistance, hypertension and fatty liver, together termed metabolic syndrome, are key risk factors for cardiovascular disease. Chronic feeding of a diet high in saturated fats and simple sugars, such as fructose and glucose, induces these changes in rats. Naturally occurring compounds could be a cost-effective intervention to reverse these changes. Flavonoids are ubiquitous secondary plant metabolites; naringin gives the bitter taste to grapefruit. This study has evaluated the effect of naringin on diet-induced obesity and cardiovascular dysfunction in high carbohydrate, high fat-fed rats. These rats developed increased body weight, glucose intolerance, increased plasma lipid concentrations, hypertension, left ventricular hypertrophy and fibrosis, liver inflammation and steatosis with compromised mitochondrial respiratory chain activity. Dietary supplementation with naringin (approximately 100 mg/kg/day) improved glucose intolerance and liver mitochondrial dysfunction, lowered plasma lipid concentrations and improved the structure and function of the heart and liver without decreasing total body weight. Naringin normalised systolic blood pressure and improved vascular dysfunction and ventricular diastolic dysfunction in high carbohydrate, high fat-fed rats. These beneficial effects of naringin may be mediated by reduced inflammatory cell infiltration, reduced oxidative stress, lowered plasma lipid concentrations and improved liver mitochondrial function in rats.

## 1. Introduction

Metabolic syndrome is defined as a collection of risk factors for cardiovascular disease, including abdominal obesity, hypertension, insulin resistance and fatty liver. This syndrome is associated with an increased dietary intake of saturated fats and simple sugars, including glucose and fructose [1,2]. The prevalence of metabolic syndrome is increasing throughout the world [1], suggesting that more can be done to provide affordable treatment options. These options include local natural products, as they could be readily available at low cost, with an appropriate safety profile, as they may have been available in foods for many generations. The flavonoids are ubiquitous as secondary metabolites in plants. Experimental and epidemiological data indicate that dietary flavonoids reduced the risk of coronary heart disease, diabetes and non-alcoholic fatty liver diseases [3], so these compounds are potential options for treatment of metabolic syndrome. 

Naringin (4′,5,7-trihydroxyflavone 7-rhamnoglucoside), found in grape fruit and related citrus species [3], and its colonic metabolite, naringenin, have been reported to show anti-inflammatory, antioxidant and cardioprotective activities, including lowering of blood glucose and cholesterol concentrations and improved insulin signalling [4,5,6,7,8]. Naringin upregulated peroxisome proliferator-activated receptor γ (PPARγ) [8], activated adenosine monophosphate (AMP) kinase [9] and suppressed liver fatty acid synthase, glucose-6-phosphate dehydrogenase, phosphatidate phosphohydrolase, HMG-CoA reductase and acyl CoA:cholesterol acyltransferase activities in type 2 diabetic mice [10]. 

We have reported that rats fed a high carbohydrate, high fat diet for 16 weeks developed many of the signs of metabolic syndrome in humans [11]. Further, chronic treatment with the flavonoid, rutin [12], or its aglycone, quercetin [13], reversed most of these symptoms, despite continuation of the high carbohydrate, high fat diet. The present study has determined whether administration of naringin at the same dose as rutin [12] reverses the metabolic parameters, as well as the changes in the structure and function of the heart, blood vessels, liver and kidneys in rats fed a high carbohydrate, high fat diet. We suggest that naringin has the potential to be a useful dietary supplement in the management of the signs of metabolic syndrome.

## 2. Experimental Section

### 2.1. Rats and Diets

Male Wistar rats (*n* = 36; 328 ± 1 g; 9–10 weeks old) were purchased from The University of Queensland Biological Resources facility. They were individually caged at the Faculty of Sciences Animal House at the University of Southern Queensland. All experimental protocols were approved by the Animal Experimentation Ethics Committee of the University of Southern Queensland, under the guidelines of the National Health and Medical Research Council of Australia. The rats were randomly divided into four separate groups: cornstarch (C; *n* = 9), C + naringin (N) (CN; *n* = 9), high carbohydrate, high fat diet (H; *n* = 9) and H + N (HN; *n* = 9). C and H rats were fed their respective diets throughout the 16-week protocol (Table 1). Drinking water in the H diet-fed rats was augmented with 25% fructose. The H diet was prepared by thoroughly mixing beef tallow, condensed milk, powdered rat feed (meat-free rat and mouse feed, Specialty Feeds) and Hubble, Mendel and Wakeman salt mixture (MP Biochemicals) [11]. In the C diet, fructose and condensed milk were replaced with corn starch (575 g) and beef tallow was replaced with water (200 mL). N was thoroughly mixed with both C and H diets at 1.6 g/kg to give an approximate dose of 100 mg/kg/day; N was administered for 8 weeks starting 8 weeks after the initiation of either diet. 

Organ weights, metabolic measurements (plasma glucose, insulin and lipid concentrations, abdominal circumference and abdominal fat pads), cardiovascular evaluations (systolic blood pressure, echocardiography, left ventricular stiffness in the Langendorff heart preparation, thoracic aortic ring reactivity, collagen deposition and inflammatory cell infiltration) and liver measurements (plasma enzymes, liver structure) were determined, as described previously [11,12,13,14]. Malondialdehyde concentrations were measured as thiobarbituric acid reactive substances by UV spectrophotometry [15].

### 2.2. Mitochondrial Preparations

Mitochondria were isolated from the liver of rats according to standard differential centrifugation procedures [16] and prepared in a medium containing 200 mM sucrose, 1 mM ethylene glycol tetraacetic acid (EGTA) and 10 mM Tris-HCl (pH 7.2). Mitochondrial protein content was determined by the bicinchoninic acid protein assay method, with bovine serum albumin (BSA) as the standard. Mitochondria were incubated at a final concentration of 0.35 mg/ml in a Clark-type electrode chamber for oxygen consumption measurements. All experiments were performed at 37 °C in a buffer containing 125 mM KCl, 1 mM EGTA, 1 mM KH_2_PO_4_ and 10 mM Tris-HCl (pH 7.2). The control (non-phosphorylating) state of respiration was initiated by the addition of 5 mM succinate/5 µM rotenone. State 3 (phosphorylating respiration) was obtained after the addition of 1 mM adenosine diphosphate (ADP). State 4 respiration was considered as the respiratory state in the absence of ADP. The efficiency of the mitochondrial oxidative phosphorylation was assessed by the state 3 to state 4 ratio, the respiratory control ratio (RCR).

### 2.3. Histology

Immediately after euthanasia, the heart and liver were blotted, weighed and cut into 3–4 mm slices, then fixed in 10% buffered formalin for 3 days, with a change of formalin solution every day to remove traces of tissue debris. The samples were then dehydrated and embedded in paraffin wax. Thin sections (5 µm) were cut and stained with either haematoxylin and eosin stain for determination of inflammatory cell infiltration and general architecture of the tissues or picrosirius red stain in the left ventricle or Milligan’s trichrome in the liver for collagen distribution. Collagen distribution was analysed by laser confocal microscopy (Zeiss LSM 510 upright Confocal Microscope). Colour intensity was quantitated using NIH-ImageJ free software (NIH) to determine the extent of collagen deposition in selected tissue sections.

### 2.4. Statistical Analysis

All data sets were represented as the mean ± standard error of mean (SEM). Comparisons of findings between groups were made via statistical analysis of data sets using one-way and two-way analysis of variance (ANOVA). When interaction and/or the main effects were significant, means were compared using Newman-Keuls multiple-comparison *post-hoc* test. A *p*-value of <0.05 was considered as statistically significant. All statistical analyses were performed using Graph Pad Prism version 5.00 for Windows.

## 3. Results

### 3.1. Metabolic Parameters

Food intake was higher in the C group compared to the H group, but energy intake was higher in H rats. This increase was associated with an increased final body weight, abdominal circumference and abdominal fat deposition compared with C rats (Table 1). Based on daily food measurements, N intake was 115.4 ± 1.9 mg/kg/day (*n* = 9) in CN rats and 95.4 ± 2.2 mg/kg/day in N rats. N did not alter the food and energy intake in either C or H rats, and body weight in CN and HN rats was not different from C and H rats, respectively (Table 1). N reduced retroperitoneal abdominal fat deposition and attenuated the increased abdominal circumference in H rats without changing omental or epididymal fat pads (Table 1). 

Basal blood glucose and plasma total cholesterol, triglyceride and non-essential fatty acid (NEFA) concentrations were increased in H diet-fed rats compared to C group rats (Table 2). N supplementation to H rats for the final eight weeks reduced plasma lipid concentrations (Table 2) and improved oral glucose tolerance (Table 1). Insulin concentrations and pancreatic wet weights were increased in H rats compared to C rats and nomalised by N (Table 1, Table 2). 

**Table 1:** Naringin on body weights, food and water intakes and organ wet weights.

Parameters	C	CN	H	HN	*p* value		
					Diet	Treatment	Interaction
Initial body weight, *g*	330 ± 3	327 ± 2	327 ± 3	327 ± 2	0.56	0.56	0.56
Final body weight, *g*	451 ± 13 ^b^	472 ± 12 ^ab^	509 ± 6 ^a^	503 ± 14 ^a^	0.0006	0.53	0.26
% of weight gain at week 16	8.3 ± 1.1 ^b^	7.6 ± 1.5 ^b^	13.6 ± 1.6 ^a^	13.4 ± 1.5 ^a^	0.0004	0.81	0.92
Food intake at week 16, *g/day*	33.4 ± 0.7 ^a^	32.2 ± 0.4 ^a^	28.0 ± 1.1 ^b^	28.1 ± 0.8 ^b^	<0.0001	0.49	0.42
Water intake at week 16, *mL/day*	31.9 ± 3.4 ^a^	34.3 ± 0.8 ^a^	22.8 ± 1.4 ^b^	28.7 ± 0.6 ^a^	0.0005	0.37	0.37
Energy intake at week 16, *kJ/day*	407 ± 9 ^a^	396 ± 5 ^a^	590 ± 20 ^b^	603 ± 11 ^b^	<0.0001	0.94	0.35
Kidney weight, *mg/mm*	47.0 ± 1.5 ^c^	57.8 ± 1.6 ^a^	55.5 ± 2.7 ^ab^	55.1 ± 1.2 ^ab^	0.1	0.0075	0.0032
Spleen, *mg/mm*	17.1 ± 0.7	19.1 ± 0.9	20.8 ± 2.3	19.4 ± 0.6	0.14	0.82	0.21
Liver wet weight, *mg/mm*	243.5 ± 5.4 ^a^	297.9 ± 11.5 ^c^	339.0 ± 14.2 ^b^	289.7 ± 8.5 ^c^	0.0002	0.81	<0.0001
Abdominal fat pads, *mg/mm*	449.2 ± 37.3 ^b^	382.0 ± 46.1 ^b^	730.3 ± 34.3 ^a^	695.8 ± 70.0 ^a^	<0.0001	0.31	0.74
Retroperitoneal fat, *mg/mm*	266.0 ± 21.9 ^b^	188.2 ± 21.3 ^c^	422.9 ± 18.7 ^a^	350.8 ± 37.0 ^a^	<0.0001	0.0072	0.92
Epididymal fat, *mg/mm*	97.4 ± 13.4 ^b^	97.5 ± 11.3 ^b^	173.4 ± 15.7 ^a^	167.7 ± 15.2 ^a^	<0.0001	0.84	0.84
Omental fat, *mg/mm*	85.8 ± 6.7 ^b^	96.2 ± 15.2 ^b^	134.0 ± 12.8 ^b^	177.3 ± 21.2 ^a^	0.0002	0.08	0.28
Abdominal circumference at 16 weeks, *cm*	21.7 ± 0.3 ^bc^	21.2 ± 0.3 ^c^	23.8 ± 0.15 ^a^	22.3 ± 0.2 ^b^	<0.0001	0.0004	0.05
Pancreas wet weight, *mg/mm*	45.3 ± 1.9 ^b^	47.0 ± 3.9 ^b^	60.8 ± 2.9 ^a^	50.0 ± 0.4 ^b^	0.0014	0.09	0.0239
**Oral glucose tolerance test (area under curve)**							
Area under curve (AUC) at 0 week	683 ± 21	679 ± 19	671 ± 18	684 ± 20	0.85	0.84	0.68
AUC at 8 weeks	682 ± 12 ^a^	700 ± 12 ^a^	802 ± 19 ^b^	781 ± 16 ^b^	<0.0001	0.91	0.21
AUC at 16 weeks	707 ± 8 ^b^	687 ± 21 ^b^	854 ± 18 ^a^	709 ± 18 ^b^	<0.0001	<0.0001	0.0011

Data are presented as the mean ± SEM, *n* = 8–9. C = cornstarch; CN = C + naringin (N); H = high carbohydrate, high fat diet; HN = H + N; AUC (area under curve) is the value under the blood glucose curve from 0 to 120 min after glucose administration. Means without a common letter in rows are significantly different at *p* < 0.05.

**Table 2:** Naringin on plasma parameters in C and H-diet fed rats.

Parameters	C	CN	H	HN	*p* values		
					Diet	Treatment	Interaction
ALT, *U/L*	28.4 ± 4.1 ^b^	33.8 ± 1.0 ^ab^	41.9 ± 2.1 ^a^	37.5 ± 2.3 ^ab^	0.0028	0.85	0.07
AST, *U/L*	68.6 ± 3.5 ^b^	67.8 ± 4.1 ^b^	84.4 ± 5.9 ^a^	72.8 ± 2.7 ^ab^	0.0201	0.15	0.21
Triglycerides, *mmol/L*	0.6 ± 0.1	1.2 ± 0.2	1.4 ± 0.4	1.6 ± 0.3	0.0369	0.16	0.47
Total cholesterol, *mmol/L*	1.6 ± 0.1 ^b^	1.7 ± 0.1 ^b^	2.2 ± 0.1 ^a^	1.6 ± 0.1 ^b^	0.0186	0.0186	0.0016
NEFA, *mmol/L*	1.4 ± 0.2 ^b^	0.7 ± 0.1 ^b^	3.8 ± 0.7 ^a^	0.8 ± 0.1 ^b^	0.0022	<0.0001	0.0044
Uric acid, *mmol/L*	46.7 ± 1.8 ^a^	29.6 ± 2.1 ^b^	49.0 ± 2.1 ^a^	43.9 ± 5.9 ^a^	0.022	0.0031	0.09
Insulin, *μmol/L*	1.9 ± 0.2 ^b^	2.2 ± 0.5 ^b^	4.2 ± 0.8 ^a^	1.7 ± 0.2 ^b^	0.08	0.0337	0.0083
Plasma TBARS, *µmol/L*	12.4 ± 1.3 ^b^	14.4 ± 0.9 ^b^	37.4 ± 6.1 ^a^	17.1 ± 1.8 ^b^	0.0097	0.0004	0.0025

Data are presented as the mean ± SEM, *n* = 6–8. ALT = alanine transaminase; AST = aspartate transaminase; TBARS = thiobarbituric acid reactive substance. Means without a common letter in rows are significantly different at *p* < 0.05.

### 3.2. Cardiovascular Structure and Function

**Figure 1:**
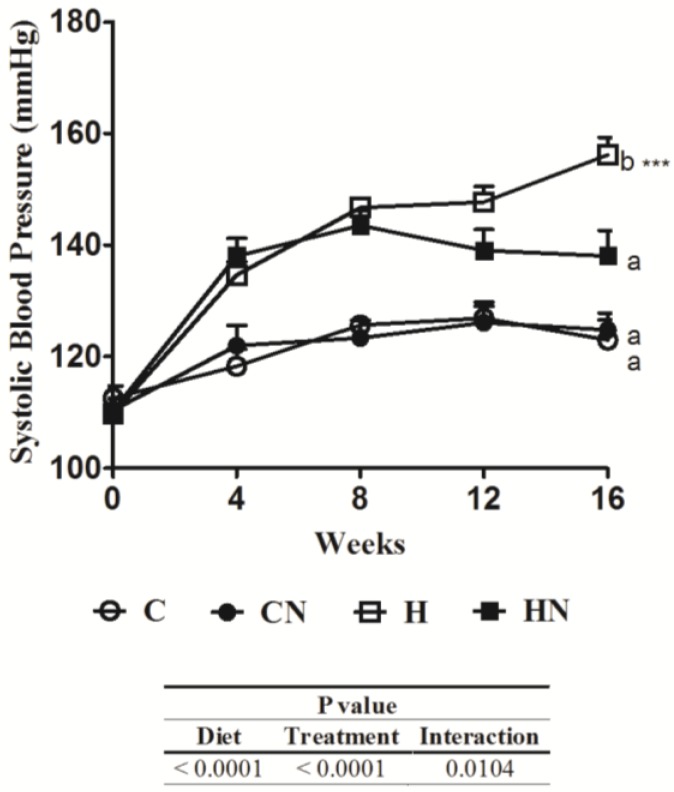
Effect of naringin on systolic blood pressure. Time course of tail cuff systolic blood pressure of Wistar rats given C, CN, H and HN (*n* = 9 in each group). Means without a common letter are significantly different at *p* < 0.05.

Systolic blood pressure was increased in the H group compared to C group rats. N reduced the systolic blood pressure in H rats (Figure 1). Echocardiographic assessment of H rats showed increased left ventricular internal diameter in diastole (LVIDd), reduced fractional shortening and ejection fraction, created greater relative wall thickness, increased estimated left ventricle (LV) mass and reduced E:A ratio (early:late (or atrial) flow through mitral valve) compared with the C group (Table 3). N normalised the LVIDd in H diet fed rats and improved fractional shortening without changing ejection fraction, relative wall thickness or E:A ratio (Table 3). *Ex vivo* cardiac function measured in the Langendorff isolated heart preparation showed markedly increased cardiac stiffness in H rats compared to C group rats that was normalised by N (Table 3). H diet-fed rats developed increased LV wet weight compared to C rats that were normalised by N (Table 3). Neither diet nor treatment affected the right ventricle (RV) wet weight (Table 3). 

**Table 3:** Naringin on cardiovascular parameters.

Parameters	C	CN	H	HN	*p* value		
					Diet	Treatment	Interaction
LVIDd, *mm*	7.14 ± 0.16 ^b^	7.59 ± 0.18 ^ab^	8.35 ± 0.26 ^a^	7.62 ± 0.21 ^ab^	<0.0001	<0.0001	<0.0001
LVPWd, *mm*	1.84 ± 0.03 ^b^	1.94 ± 0.12 ^b^	2.22 ± 0.05 ^a^	1.92 ± 0.05 ^b^	0.0215	0.18	0.0116
Fractional shortening, *%*	48.2 ± 1.2 ^a^	56.4 ± 4.9 ^a^	35.7 ± 1.2 ^b^	48.3 ± 2.8 ^a^	0.0021	0.002	0.47
Ejection fraction, *%*	90.7 ± 1.2 ^b^	89.4 ± 1.5 ^b^	96.5 ± 0.3 ^a^	92.5 ± 1.3 ^b^	0.001	0.0312	0.26
Relative wall thickness	0.49 ± 0.01 ^a^	0.57 ± 0.06 ^b^	0.49 ± 0.01 ^a^	0.50 ± 0.02 ^a^	0.08	0.0296	0.08
Ascending aortic flow, *m/s*	0.86 ± 0.02	0.98 ± 0.13	1.08 ± 0.05	0.82 ± 0.08	0.7	0.37	0.0203
Descending aortic flow, *m/s*	0.79 ± 0.6	0.73 ± 0.8	0.96 ± 0.7	0.77 ± 0.7	0.15	0.09	0.36
E:A ratio	1.9 ± 0.2	- *	1.4 ± 0.2	1.7 ± 0.4	-	-	-
Ejection time, *ms*	87.3 ± 1.5 ^a^	69.3 ± 8.6 ^b^	77.0 ± 2.8 ^ab^	81.8 ± 2.7 ^ab^	0.81	0.16	0.0187
Deceleration time, *ms*	58.5 ± 1.9 ^a^	37.9 ± 3.6 ^b^	45.4 ± 2.9 ^b^	47.0 ± 3.8 ^b^	0.06	0.0472	0.0151
Estimated LV mass, *g*	0.84 ± 0.04 ^b^	1.11 ± 0.09 ^ab^	1.25 ± 0.09 ^a^	1.01 ± 0.05 ^ab^	0.0411	0.84	0.0017
Heart wet weight, *mg/mm*	26.5 ± 0.9 ^b^	25.4 ± 0.6 ^b^	30.1 ± 1.4 ^a^	25.6 ± 0.5 ^b^	0.05	0.0055	0.08
LV wet weight, *mg/mm*	20.5 ± 0.4 ^b^	18.8 ± 0.5 ^b^	22.7 ± 0.8 ^a^	19.8 ± 0.4 ^b^	0.007	0.0002	0.29
RV wet weight, *mg/mm*	4.7 ± 0.2	4.4 ± 0.2	4.6 ± 0.2	4.3 ± 0.2	0.62	0.14	1
LV stiffness constant, *κ*	22.9 ± 0.4 ^b^	24.6 ± 0.9 ^b^	29.3 ± 0.5 ^a^	25.1 ± 1.0 ^b^	<0.0001	0.11	0.0005
LV interstitial collagen, *%*	5.8 ± 0.5 ^b^	4.8 ± 0.9 ^b^	18.4 ± 0.8 ^a^	13.5 ± 0.5 ^b^	0.0212	0.0025	<0.0001

* Data not available; LVIDd = left ventricular internal diameter in diastole; LVPWd = left ventricular posterior wall thickness in diastole; LV = left ventricle; RV = right ventricle; E:A = early:late (or atrial) velocity through the mitral valve. Data are presented as the mean ± SEM, *n* = 8–9. Means without a common letter in rows are significantly different at *p* < 0.05.

**Figure 2:**
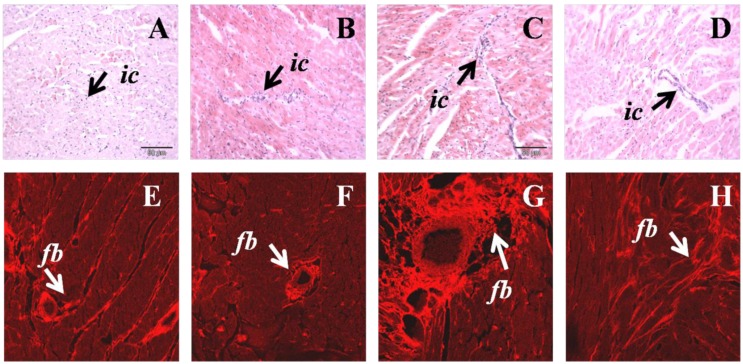
Hematoxylin and eosin staining of left ventricular tissue showing inflammatory cell infiltration of C (**A**), CN (**B**) H (**C**), HN (**D**) (upper panel); *ic*, inflammatory cell. Picrosirius red staining for left ventricular tissue collagen of C (**E**), CN (**F**), H (**G**), HN (**H**); *fb*, collagen deposition (lower panel).

Increased infiltration of inflammatory cells was observed in H left ventricle, together with increased collagen deposition compared to C rats (Figure 2). H diet-fed rats showed hypertrophy of cardiomyocytes that was reduced following N treatment (Figure 2). N normalised inflammatory cell infiltration and markedly reduced collagen deposition in H diet-fed rats (Figure 2).

**Figure 3:**
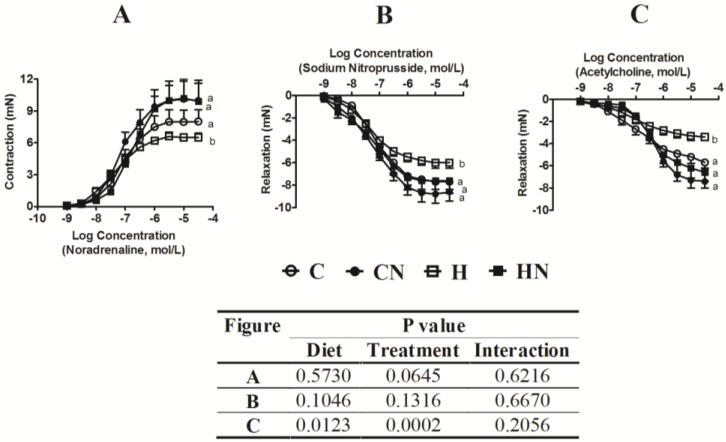
Cumulative concentration-response curves for noradrenaline (**A**), sodium nitroprusside (**B**) and acetylcholine (**C**) in thoracic aortic rings derived from C, CN, H and HN treated groups. All concentrations are expressed as log concentration (mol/L). Data are shown as the mean ± SEM, *n* = 8.

H feeding in rats diminished vascular responses in isolated thoracic aortic rings to noradrenaline, sodium nitroprusside and acetylcholine compared to C rats (Figure 3). Administration of N to H rats improved contraction mediated by noradrenaline, as well as relaxation mediated by sodium nitroprusside and acetylcholine in aortic rings compared to H rats (Figure 3). Administration of N to C rats did not change responses (Figure 3).

### 3.3. Liver Structure and Function

**Figure 4:**
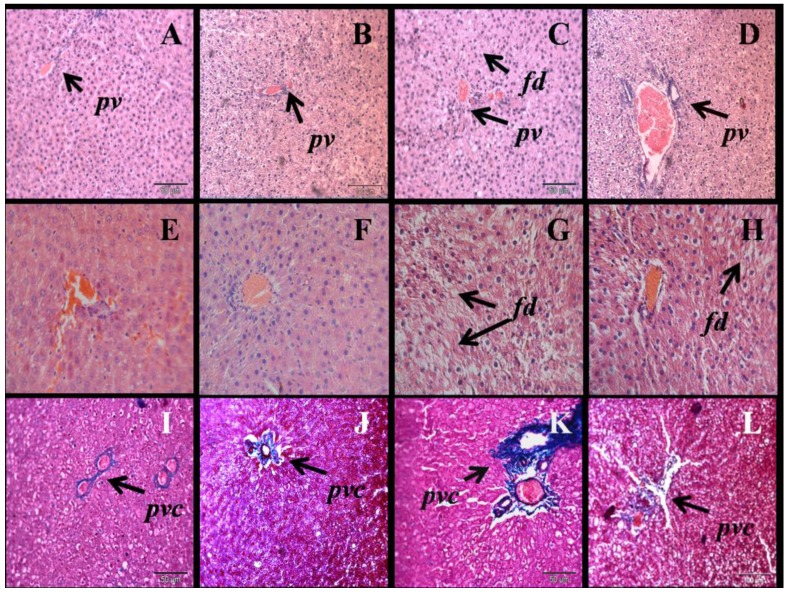
Haematoxylin and eosin staining of liver (×20) showing fat deposition in C (**A**), CN (**B**) H (**C**), HN (**D**) rats (*fd*, fat droplets; *pv,* portal vein (upper panel)) and ×40 for C (**E**), CN (**F**) H (**G**), HN (**H**) rats (middle panel). Milligan’s trichrome staining of the liver section (×20) showing collagen as darker blue region in C (**E**), CN (**F**), H (**G**), HN (**H**) rats; *pvc*, portal vein collagen (lower panel).

H rats had increased liver wet weight compared to C rats (Table 1). N normalised liver wet weight in H rats. H rats showed increased infiltration of inflammatory cells and increased deposition of collagen around the blood vessels in liver sections compared to C rats (Figure 4). N reduced inflammation and decreased collagen deposition in the liver of H rats. H rats increased deposition of fat droplets in liver, which was reduced by N (Figure 4). H rats increased plasma activity of aspartate aminotransferase (AST) and alanine aminotransferase (ALT) compared to C rats; the increases were normalised by N (Table 2).

Mitochondrial respiration (state 3, state 4 and uncoupled respiration rates, respiratory control ratio (RCR)) examined in isolated liver mitochondria using the flavin adenine dinucleotide (FAD)-linked substrate, succinate, showed decreased state 3 respiration in H rats (Table 4). Consequently, RCR was decreased for succinate plus rotenone (Table 4). N normalised state 3 respiration in H rats (Table 4). The high RCR values (Table 4) suggest that the structural and functional integrity of the mitochondrial preparations was preserved throughout these experiments. 

**Table 4:** Succinate-dependent respiratory parameters of isolated liver mitochondria.

Respiratory parameter	C	CN	H	HN	*p* Value		
					Diet	Treatment	Interaction
Succinate + Rotenone-dependent							
State 3, *nmol/min/mg protein*	121.8 ± 15.6 ^a^	120.6 ± 17.3 ^a^	55.7 ± 6.6 ^b^	111.4 ± 2.8 ^a^	0.0069	0.0394	0.0324
State 4, *nmol/min/mg protein*	19.2 ± 3.2	22.8 ± 2.0	17.4 ± 1.2	26.7 ± 3.4	0.71	0.0343	0.32
RCR	6.8 ± 1.0 ^a^	5.6 ± 1.1 ^ab^	3.2 ± 0.3 ^b^	4.5 ± 0.6 ^ab^	0.0108	0.95	0.14

Data are presented as the mean ± SEM, *n* = 3–5. RCR = respiratory control ratio. Means without a common letter in rows are significantly different at *p* < 0.05.

## 4. Discussion

Chronic feeding of a diet rich in simple carbohydrates and saturated fats induces the signs of metabolic syndrome in rats [11], with some or all signs being reversed when natural compounds from foods, including flavonoids, such as rutin and quercetin, are added to the diet [12,13]. This study showed that naringin produces similar responses to rutin and quercetin in high carbohydrate, high fat-fed rats. Our results suggest that several mechanisms may contribute to the reversal of signs, including reduced inflammatory cell infiltration, reduced oxidative stress, lowered plasma lipid concentrations and improved liver mitochondrial function. 

Metabolic syndrome is a collection of risk factors for cardiovascular disease. Naringin showed a range of properties that help protect the cardiovascular system, including antihypertensive, lipid-lowering, insulin-sensitising, anti-oxidative and anti-inflammatory properties [17]. Naringin prevented the age-related increase in systolic blood pressure in stroke-prone spontaneously hypertensive rats, increased nitric oxide production, improved endothelial function and decreased cerebral thrombotic tendency [18]. Further, naringin prevented oxidative stress in the hearts of rats with isoprenaline-induced myocardial infarction [19]. 

Obesity, an important component of metabolic syndrome, is a chronic low-grade inflammatory condition leading to adipocyte differentiation and growth in adipose tissues [20]. In mice fed a high fat diet, naringin decreased visceral adiposity and lowered plasma lipid concentrations, probably by activation of AMP kinase [9]. Naringin was more effective as an anti-inflammatory compound than indomethacin in the air pouch model of inflammation [21]. The increased production of inflammatory cytokines in rats treated with streptozotocin and a high fat diet was reversed by chronic treatment for four weeks with naringin (50 mg/kg) [22]. Naringenin, the aglycone of naringin produced by colonic bacteria before absorption, induced growth arrest, apoptosis and lipolysis in a cell line of human pre-adipocytes, in contrast to naringin [23]. In the high carbohydrate, high fat dietary model, we have shown that both the closely related flavonoid, rutin, and its aglycone, quercetin, decreased abdominal fat pads [12,13], similar to naringin in this study. 

Insulin resistance/hyperglycaemia and hyperlipidaemia are two of the diagnostic criteria for metabolic syndrome. In type 2 diabetic *db*/*db* mice, naringin prevented the progression of hyperglycaemia by increasing hepatic glycolysis and lowering hepatic gluconeogenesis [24]. Naringin (40 mg/kg in rats) effectively inhibited dipeptidyl peptidase-IV *in vivo*, lowering blood glucose and increasing blood insulin concentrations [25]. Regulation of glucose-regulating enzymes, such as phosphoenolpyruvate carboxykinase and glucose-6-phosphotase following activation of AMP kinase by naringin, reduced plasma blood glucose concentrations in mice fed a high fat diet [9]. In these high fat-fed mice, naringin lowered lipid synthesis, increased fatty acid oxidation, reversed adipocyte hypertrophy and decreased hepatic steatosis [9]. Further, naringin lowered cholesterol biosynthesis in rats fed a high fat and high cholesterol diet [26]. However, treatment of moderately hypercholesterolaemic men and women with naringin 500 mg/day did not change plasma cholesterol concentrations [27], showing the difficulty of extrapolating rat results to humans. 

Liver function may be improved in naringin-treated rats by decreased lipid peroxidation in diabetes [28], improvement of hepatic insulin signalling by naringenin [7] and improved liver structure and function, as with the closely related flavonoids, rutin [12] and quercetin [13]. Liver mitochondrial function was compromised in the liver of high fat-fed rats [29]. Transcriptional regulators of mitochondria are important in lipid metabolism and steatosis in liver. Naringin increased PPARγ protein expression, a transcriptional regulator of mitochondrial function, and prevented steatosis in diabetic male rats fed a high fat diet, probably by decreasing the expression of liver X receptor (LXR) and sterol regulatory element-binding protein (SREBP)-1c and SREBP-1a in liver [8]. In our study, naringin improved the respiratory function of isolated mitochondria from liver, suggesting improvement of mitochondrial function and lipid metabolism.

The low water solubility limits the bioavailability of flavonoids that depend upon the efficiency of their transfer through the brush border and the capacity of the intestine to secrete conjugated metabolites [30]. The colon microflora hydrolysed naringin to naringenin, which is then absorbed from the colon to increase apparent bioavailability [31]. The ingestion of naringenin from capsules or naringin from grapefruit juice (135–199 mg naringenin equivalent) led to peak plasma concentrations of 6.0–7.3 μmol/L [32,33]. Commercial grapefruit juice ingestion in healthy volunteers showed peak plasma concentrations ranging from 0.3 to 1.5 μmol/L [34]. 

One potential mechanism of action of polyphenols, such as naringin and naringenin, involves changes in the gastrointestinal microbial population. This population has been implicated in human disease states, including obesity [35], with polyphenols being both substrates for microbial biochemical pathways and modulators of bacterial growth [36]. Flavonoid aglycones, such as naringenin and quercetin, inhibited the growth of a wide range of gastrointestinal bacteria to a much greater extent than the parent glycosides [37]. These mechanisms open up a wide range of therapeutic possibilities for natural plant products as part of the diet [38]. 

In this study, rats were administered naringin at a dose of approximately 100 mg/kg/day. This dose corresponds to ~1 g/day naringin in a 70 kg human based on scaling equations [39] or ~0.6 g/day based on body surface area comparisons between rats and humans [40]. Although the average daily human intake of naringin is not known, the total intake of polyphenols is ~1 g/day, with two-thirds being flavonoids [41]. This suggests that the dose of naringin used in this study is realistic in humans. This could also suggest that the dose of 500 mg/day in hypercholesterolaemic patients [27] may be too low to achieve full responses. Naringin is listed as “generally regarded as safe”, and chronic effects at doses of 1 g/day and greater have not been reported. Naringin may need to be given in divided doses, since the half-life of the aglycone, naringenin, produced in the distal colon, was reported as 2.3 h in humans [33].

## 5. Conclusions

Naringin was effective against the symptoms of metabolic syndrome in a diet-induced obese rat model. Naringin decreased inflammatory cell infiltration, lowered plasma lipids, improved oxidative stress and mitochondrial function as the probable mechanisms to reverse metabolic syndrome. Thus, citrus flavonoids, such as naringin, can be considered as a therapeutic option for the treatment of metabolic syndrome; clinical trials are required to establish the safety of these natural compounds.
